# Systematic reviews and meta-analyses addressing comparative test accuracy questions

**DOI:** 10.1186/s41512-018-0039-0

**Published:** 2018-09-10

**Authors:** Mariska M. G. Leeflang, Johannes B. Reitsma

**Affiliations:** 1 Department Clinical Epidemiology, Biostatistics and Bioinformatics, Amsterdam Public Health, Amsterdam UMC, University of Amsterdam, Amsterdam, The Netherlands; 2 Julius Center for Health Sciences and Primary Care, University Medical Center Utrecht, Utrecht, The Netherlands

**Keywords:** Meta-analysis, Systematic reviews, Diagnostic test accuracy, Comparative accuracy

## Abstract

**Background:**

While most relevant clinical questions are comparative, most diagnostic test accuracy studies focus on the accuracy of only one test. If we combine these single-test evaluations in a systematic review that aims to compare the accuracy of two or more tests to indicate the most accurate one, the resulting comparative accuracy estimates may be biased.

**Methods and results:**

Systematic reviews comparing the accuracy of two tests should only include studies that evaluate both tests in the same patients and against the same reference standard. However, these studies are not always available. And even if available, they may still be biased. For example because they included a specific patient group that would not have been tested with two or more tests in actual practice. Combining comparative and non-comparative studies in a comparative accuracy meta-analysis requires novel statistical approaches.

**Conclusion:**

In order to improve decision-making about the use of test in practice, better designed and reported primary diagnostic studies are needed. Meta-analytic and network-type approaches available for therapeutic questions need to be extended to comparative diagnostic accuracy questions.

## Background

A central question in clinical epidemiology is: “compared to what?”. Aspirin may be beneficial against headache, but compared to what? If 50% of the patients with episodic headache benefit from taking an aspirin, we also need to know whether 50% would have been relieved without any treatment or with another treatment as well.

Unfortunately, if we turn to medical test evaluations, a large number of studies focus on the accuracy of a single test [1]. This implies that we are able to judge a medical test purely on its own. Whether a sensitivity of 70% suffices to use a test in practice depends on the seriousness of the disease, and especially on the consequences associated with its false negative results, but it ignores the fact that existing tests may also be able to detect 70% of the patients with the disease of interest. For many diseases, this has led to a large number of different tests and biomarkers that have all been evaluated on their own, resulting in the conclusion that the test could be useful in practice, but overlooking how each test relates to its competitors. Indeed, these tests may have been evaluated against a reference standard, necessary to determine sensitivity and specificity, but this reference standard will often not be a realistic alternative for the other test. The accuracy of the test of interest should be compared to the accuracy of other relevant tests that are a realistic alternative. This problem of inappropriate test comparators is then further perpetuated in systematic reviews of diagnostic accuracy. In November 2017, the Cochrane Library contained 88 diagnostic test accuracy reviews, of which 52 indeed address a comparative question [2]. However, more than two thirds of the included primary studies only focused on one of the tests of interest for the review. But if the studies evaluating the accuracy of test A have been done in a different patient population than the studies evaluating test B, then we will never be able to know whether any difference we find between the tests can be contributed to the tests or is the result of other factors that differ between studies, such as study setting or population [3]. Even if the relevance of comparative accuracy is apparent to the review authors, actually addressing the question in a comparative way is limited by the available evidence base.

### Comparative test accuracy

For the diagnosis of Lyme disease, some laboratories provide a positive test result based on only one serological test, while others use a two-tiered testing approach in which the test positives on the first test are retested with a second, different test. Which approach leads to a higher overall accuracy? In another scenario, internal medicine specialists may wonder if they should use ultrasound or CT scanning before referring a patient for surgery for suspected appendicitis. Primary studies as well as systematic reviews only focusing on one of these tests lack clinically relevant information.

In a primary study, the accuracy of two tests may be compared in different ways [1, 4]. In the case of laboratory tests, it may be feasible to apply all relevant tests and the reference standard to the same patient. Such a design provides us with a direct comparison between the different tests of interest and seems to be the option with the lowest risk of bias. However, in some cases, such as when comparing the accuracy of CT with the accuracy of MRI, it may not be feasible or ethical to submit all participants to three potentially burdensome techniques. Randomisation may be a solution in such a situation, although the disadvantage there is that it will not allow for the possibility of comparing results of patients whose CT and MRI results disagree. The third, and least preferable way to compare the accuracy of two tests, is to apply these tests to different participants, according to the judgement of the researcher or based on previous test results.

### Only include the unbiased studies?

In an ideal world, all systematic reviews that compare the accuracy of two tests should only include studies that evaluate both tests in the same patients and against the same reference standard. However, of the 52 comparative accuracy reviews in the Cochrane Library, only 22 included more than three primary studies directly comparing the accuracy of two index tests. If we would include only primary studies with a comparative design, then we would end up with numerous “empty” reviews. Besides, for many diseases, we often have an array of different tests available. Hence, authors of systematic reviews may wish to not only compare the accuracy of one test versus the accuracy of another, but in some cases aim to select the most accurate test from a set of available tests. Although for some in vitro tests it may be easier to have a number of tests done on the same patient sample, there are still many other tests that we will never be able to make all possible comparisons. We may therefore need to accept that single-test studies may remain a valuable source of evidence.

Another reason why solely focusing on comparative accuracy studies may not be straightforward is that we are not sure whether these designs really provide us with the least biased or the most applicable comparative accuracy estimate. The studies evaluating multiple tests may have included a skewed population of patients for whom it was necessary to use more than one test to come to a diagnosis, while the review question is really about one test or the other. However, we do not yet have a validated tool to assess both the risk of bias and concerns for applicability for a comparative accuracy study. So the review author stating a clinically relevant comparative question ends up with a mix of single-test studies and comparative studies and has to find out for him or herself how to tailor the Quality Assessment for Diagnostic Accuracy Studies (QUADAS-2) tool for the comparative question. For example, a signalling question about providing the same clinical information to the assessors of all tests may be added, and whether all study participants received all tests [5].

### Possible solutions?

Methodological development should therefore focus on ways to combine comparative and non-comparative studies in comparative meta-analyses. One approach may be to combine comparative studies with those single-test studies that appear to be least biased or most representative. Better adherence to the STAndards for Reporting Diagnostic accuracy studies (STARD) is needed to enable selection of the “better” studies, as well as a deeper understanding of factors influencing the choice of tests and comparability of tests. This requires a more solid knowledge of the data at hand, asking for individual patient data analyses and additional information about test usage, i.e. what drives the choice for one test over another. Although STARD does not specifically focus on test comparisons, it does mention that a study can “evaluate the accuracy of one or more index tests” [6].

Combining comparative and non-comparative studies in a comparative accuracy review provides review authors with a mix of designs and data-structures. Taking these different data-structures (e.g. paired data versus single-test data) into account in a meta-analysis requires new statistical approaches. At the moment, these methods are still under development. They can be roughly divided into two groups: arm-based comparisons, which compare the summary estimates of one test with the summary estimates of the other test [7–9], and contrast-based approaches, which first estimate the difference in accuracy between the two tests per study and then meta-analyses these differences [10]. Some of these methods can also incorporate the data from single-test studies [7, 8, 10] and some cannot [9]. All models claim that they can be extended for more than two tests, although none of the reports clearly illustrate this, and all models are relatively complicated, using Bayesian statistics or copula methodology. The next step is to investigate to what extent they outperform straightforward meta-regression with different test-types as covariate.

### Beyond diagnostic accuracy

The problem of focussing on a single test in diagnostic test research is not unique. For example, a recent review revealed 125 studies presenting 363 different models for cardiovascular disease, a number which in itself makes it nearly impossible to compare all available models [11]. However, even if all future studies would compare all clinically relevant scenarios in terms of accuracy or prognostic performance, then we may be still missing a part of the evidence puzzle that is needed to make decisions about medical tests and biomarkers. Just the accuracy or prognostic performance of a test says nothing about whether the use of the test or marker will in the end improves patient outcomes. This refers to a different level of comparisons between tests: the comparison of two tests in terms of effectiveness or clinical utility.

## Conclusion

In order to improve decision-making about the use of test in practice, several advancements in diagnostic research are necessary. It starts with better designed and reported primary diagnostic studies. Too frequently, the focus is on the evaluation of a single test, often using retrospective data on convenient samples which are fraught with problems. Meta-analytic and network-type approaches available for therapeutic questions need to be extended to comparative diagnostic accuracy questions.

## Abbreviation

QUADAS: Quality assessment of diagnostic accuracy studies
